# Beef Tenderness Prediction by a Combination of Statistical Methods: Chemometrics and Supervised Learning to Manage Integrative Farm-To-Meat Continuum Data

**DOI:** 10.3390/foods8070274

**Published:** 2019-07-22

**Authors:** Mohammed Gagaoua, Valérie Monteils, Sébastien Couvreur, Brigitte Picard

**Affiliations:** 1 UMR Herbivores, VetAgro Sup, Université Clermont Auvergne, INRA, F-63122 Saint-Genès-Champanelle, France; 2 URSE, Ecole Supérieure d’Agriculture (ESA), Université Bretagne Loire, 55 Rue Rabelais, BP 30748, 49007 Angers, CEDEX, France

**Keywords:** beef tenderness, machine learning, farm-to-fork, carcass, rearing practices, decision trees, cows

## Abstract

This trial aimed to integrate metadata that spread over farm-to-fork continuum of 110 Protected Designation of Origin (PDO)Maine-Anjou cows and combine two statistical approaches that are chemometrics and supervised learning; to identify the potential predictors of beef tenderness analyzed using the instrumental Warner-Bratzler Shear force (WBSF). Accordingly, 60 variables including WBSF and belonging to 4 levels of the continuum that are farm-slaughterhouse-muscle-meat were analyzed by Partial Least Squares (PLS) and three decision tree methods (C&RT: classification and regression tree; QUEST: quick, unbiased, efficient regression tree and CHAID: Chi-squared Automatic Interaction Detection) to select the driving factors of beef tenderness and propose predictive decision tools. The former method retained 24 variables from 59 to explain 75% of WBSF. Among the 24 variables, six were from farm level, four from slaughterhouse level, 11 were from muscle level which are mostly protein biomarkers, and three were from meat level. The decision trees applied on the variables retained by the PLS model, allowed identifying three WBSF classes (Tender (WBSF ≤ 40 N/cm^2^), Medium (40 N/cm^2^ < WBSF < 45 N/cm^2^), and Tough (WBSF ≥ 45 N/cm^2^)) using CHAID as the best decision tree method. The resultant model yielded an overall predictive accuracy of 69.4% by five splitting variables (total collagen, µ-calpain, fiber area, age of weaning and ultimate pH). Therefore, two decision model rules allow achieving tender meat on PDO Maine-Anjou cows: (i) IF (total collagen < 3.6 μg OH-proline/mg) AND (µ-calpain ≥ 169 arbitrary units (AU)) AND (ultimate pH < 5.55) THEN meat was very tender (mean WBSF values = 36.2 N/cm^2^, *n* = 12); or (ii) IF (total collagen < 3.6 μg OH-proline/mg) AND (µ-calpain < 169 AU) AND (age of weaning < 7.75 months) AND (fiber area < 3100 µm^2^) THEN meat was tender (mean WBSF values = 39.4 N/cm^2^, *n* = 30).

## 1. Introduction

Among the eating qualities of meat, tenderness is often reported as one of the main drivers of beef palatability that dictates the overall liking of cooked meat or to make (re)purchasing decision [1,2,3]. However, it has been reviewed that for consumer confidence, there is need to guarantee consistent and high eating quality of meat [4]. From the large literature, there is a consensus that this is a challenging task to achieve consistent eating quality as meat is biochemically dynamic and susceptible to variation. Indeed, variations in beef tenderness stems from a wide range of factors which are intrinsic and extrinsic and measurable from the farm-to-fork continuum levels [5,6,7,8]. The modern beef industry seeks new strategies using the whole or part of these factors to develop management and predictive tools. These tools would provide products of consistent quality that meet consumer expectations, paying specific attention to sensory traits. Accordingly, we recently proposed a holistic approach that considers 4 levels of the farm-to-fork live period of the animals (farm level: rearing factors and animal characteristics, slaughterhouse level: carcass characteristics, muscle level: muscle characteristics and protein biomarkers, meat level: meat quality traits) to sufficiently characterize the driving factors in relation to different desirable qualities of meat, namely tenderness [8,9].

Therefore, we intend to use metadata that spread over this continuum, to identify how carcass and beef qualities can be jointly managed using rearing practices applied during the whole life of the animals or by a combination of proxies that belong to the other levels of the continuum [8]. To achieve this challenging objective, we proposed to implement various statistical strategies to analyze this metadata by defining three main purposes: (i) apply/develop appropriate statistical tools to relate accurately the different elements of the continuum; (ii) determine the most appropriate methods of rearing practices to meet the expectations of the slaughterers; and (iii) provide breeders/slaughterers with decision tools (predictive) for joint management of carcass and meat quality potential [10]. Hence, partial least squares regression (PLS) and decision trees were applied in this work to achieve the fixed objectives on PDO Maine-Anjou cull cows. Overall, the combined statistical techniques used in this trial showed the possibility to propose recommendations that would help make decisions about how joint management of the qualities of carcasses and their produced beef will help reach the targeted market specifications.

## 2. Materials and Methods

### 2.1. Experimental Design and Animal Characteristics and Rearing Factors

In this trial, we used the data of the same 110 PDO Maine-Anjou cows from previous experimental designs that are described in details by Gagaoua et al. [11] and Couvreur et al. [12]. The investigated cows are from a cooperative of livestock farmers located in the department of Maine-et-Loire, France. All the animals were collected and slaughtered following the same protocol and in the same commercial slaughterhouse (Elivia, Lion d’Angers, France). This collaboration allowed us collecting information on animals such the feeding regimen during the whole life of the animal as well as the day before slaughter, conditions of transport to the slaughterhouse and duration, conditions of resting period after arrival at the abattoir, conditions of resting with free access to water but food deprived, stunning procedure, as well the conditions of chilling and storing of the carcasses.

The rearing practices of each animal were obtained by a survey carried out by directly interviewing farmers and described in detail by Couvreur et al. [12]. The survey included 16 quantitative and qualitative questions (Table 1) subdivided into two categories:

(i) Questions related to the finishing period: part of hay, haylage and/or grass in the finishing diet (% *w*/*w*); daily and global amount of concentrate (kg); fattening duration (days); physical activity (% days out)
(ii) Questions related to animal characteristics: animals with beef or dairy-ability; birth month/season; birth weight (kg); age at weaning (month); duration of the period between the last weaning and the beginning of the finishing period (days); age of first calving; number of calving; suckling value (0–10) and age at slaughter.

### 2.2. Slaughtering, Carcass Characteristics and Muscle Sampling

All the cows were slaughtered using captive bolt pistol prior to exsanguination. They were dressed following the standard commercial practices in compliance with the French welfare and EU regulations (Council Regulation (EC) No. 1099/2009). The carcasses were not electrically stimulated. We chilled the carcasses during 24 h *p-m* (post mortem) at 2–3 °C. After slaughter, the carcasses were characterized and graded using the European beef grading system (CE 1249/2008). A total of 8 carcass characteristics (Table 2) were recorded: the carcass weight (kg), conformation score (1–15 scale), weight of the 5th ribeye, muscle carcass weight (g) of the 5th rib, fat carcass weight (g) of the 5th rib, fat-to-muscle ratio in the 5th rib (% *w*/*w*), color score of the carcass (1–5 scale) and tenderness score of the carcass (1–5 scale).

The *Longissimus thoracis* (LT, mixed fast oxido-glycolytic) muscle samples were removed from the right-hand side of each animal carcass 24 h *p-m* from as detailed in Gagaoua et al. [11]. Briefly, from the four parts that were taken, one was frozen in liquid nitrogen and kept at −80 °C until analyzed for muscle biochemistry by the quantification of fiber area, the percentages of myosin heavy chains isoforms (MyHC), the activities of metabolic enzymes describing both the glycolytic and oxidative pathways, biomarkers of beef tenderness quantified by the immunobased Dot-Blot technique. The second part was cut into pieces of 1–2 cm cross-section, vacuum packed and stored at −20 °C until analyzed for intramuscular fat content and intramuscular connective tissue. The third part was used to evaluate meat color coordinates and ultimate pH. The fourth part was cut into 20 mm thick steaks and vacuum packed in sealed plastic bags for 14 days ageing at 4 °C. For these aged meat samples, the steaks were frozen and stored at −20 °C until shear force measurements of tenderness.

### 2.3. Muscle Characteristics Determination

There were 30 muscle characteristics quantified from the muscle level (Table 3). The parameters corresponded to myosin fibers describing the contractile properties, oxidative and glycolytic metabolic enzyme activities to define the metabolic properties of the muscles; intramuscular connective tissue properties by collagen contents and beef tenderness protein biomarkers by their abundance [6,11].

For metabolic muscle type, we measured the activities of isocitrate dehydrogenase (ICDH; EC 1.1.1.42) and lactate dehydrogenase (LDH; EC 1.1.1.27) [13]. Both enzymes are representative of main steps of the oxidative and glycolytic pathways, respectively and are routinely used to determine the metabolic types of beef muscles [14].

The contractile properties were determined by the determination of the percentages of myosin heavy chains (MyHC) isoforms using an adequate mini-gel electrophoresis protocol [15]. Controls of bovine muscle containing 3 (MyHC-I, IIa and IIx) or 4 (MyHC-I, IIa, IIx and IIb) muscle fibers were run at the extremities of each gel [16]. Thus, 3 isoforms of MyHC isoforms were quantified. The quantification of the bands revealed no existence of MyHC-IIb isoform in PDO Maine-Anjou breed, therefore only MyHC-I, IIa and IIx isoforms are reported.

The muscle mean cross sectional fiber area for all the animals was determined on 10-μm thick sections cut perpendicular to the muscle fibers with a cryotome [13]. Between 100 and 200 fibers in each of the two different locations in the muscle were used to determine the mean fiber area (in µm^2^) by computerized image-analysis.

For total, insoluble collagen and percentage of soluble collagen, we used the frozen muscle. First, it was homogenized in a household cutter, freeze-dried for a period of 48 h before pulverization in a horizontal blade mill. Afterward, it was stored at +4°C in stopper plastic flasks until analyses. For total collagen and following our previously described protocol, about 250 mg of muscle powder were weighed and acid hydrolysed with 10 mL of 6 N HCl overnight at 110 °C in a screw-capped glass tube. The acid hydrolysate was diluted 5 times in 6 N HCl and the subsequent procedure used was as Dubost et al. [17]. For soluble/insoluble collagen, muscle powder was solubilised and hydrolysed according to the same method as for total collagen. For total and insoluble collagen, each sample was weighed and measured in duplicate and data were expressed in mg of hydroxyproline per g of dry matter (mg OH-Pro·g^−1^ DM). From the average values of these parameters and for each sample, the solubility of collagen was calculated as their ratio as following:

$$Soluble\;collagen=\;\frac{Total\;collagen-Insoluble\;collagen}{Total\;collagen}\;\times 100\%$$

The relative abundances of the 21 beef tenderness biomarkers were determined as cited above using Dot-Blot [16,18]. The quantified biomarkers belong to 6 different but interacting biological pathways:

1. heat shock proteins (αB-crystallin, Hsp20, Hsp27, Hsp40, Hsp70-1A, Hsp70-1B, Hsp70-8 and Hsp70-Grp75);
2. metabolism (Enolase 3 (ENO3) and Phosphoglucomutase 1 (PGM1));
3. structure (α-actin, Myosin binding protein H (MyBP-H), Myosin light chain 1F (MyLC-1F) and Mysoin heavy chain IIx (MyHC-IIx));
4. oxidative stress (Superoxide dismutase [Cu-Zn] (SOD1), Peroxiredoxin 6 (PRDX6) and Protein deglycase (DJ1));
5. proteolysis (µ-calpain and m-calpain);
6. apoptosis and signaling (Tumor protein p53 (TP53) and H2A Histone (H2AFX)).
7. The conditions retained and suppliers for all primary antibodies dilutions and details of the protocol are exactly the same of our previous work using the same data [11]. The relative protein abundances of the biomarkers were based on the normalized volume and expressed in arbitrary units (A.U).

### 2.4. Meat Quality Traits

At the meat level, 6 eating quality were evaluated (Table 4).

Ultimate pH (pHu) was evaluated at 24 h *p-m* in each muscle sample using a Hanna pH meter (HI9025, Hanna Instruments Inc., Woonsocket, RI, USA) suitable for meat penetration. The measurements were done by inserting a glass electrode between the 6th and 7th rib. The pH meter was calibrated at chilling temperature using standard pH 4 and pH 7 buffers.

For surface fresh meat color determination, a portable colorimeter (Minolta CR400, Konica Minolta, Japan) was used to measure L*, a* and b* coordinates as described by Gagaoua et al. [7].

For intramuscular fat (IMF) content, a Dionex ASE 200 Accelerated Solvent Extractor (Dionex Corporation, Sunnyvale, CA, USA) was used. Briefly, muscle dry matter was assayed gravimetrically after drying at 80 °C for 48 h. Then, total lipids were extracted by mixing 6 g of muscle powder with chloroform-methanol according to the method of Folch et al. [19]. Each sample was measured in triplicate and data were expressed in g per 100 g of dry matter (g/100 g DM).

For objective tenderness, Warner-Bratzler shear force (WBSF) was measured according to Lepetit and Culioli [20] using a Warner-Bratzler shear device (Synergie200 texturometer, MTS, Eden Prairie, MN, USA). After thawing 48 h at 4 °C, the steaks were placed for 4 h in a thermostated bath at 18 °C [12]. Then, they were cooked using an Infra Grills E (Sofraca, Athis-Mons, France) set at 300 °C until the temperature at the heart of the steak reached 55 °C, a usual temperature in France [21]. From 3–5 test pieces (1 × 1 × 4 cm) were taken from the heart of the steak in the direction of the fibers and 3–4 repetitions per test tube were carried out. A 1 kN load cell and a 60 mm/min crosshead speed were used (universal testing machine, MTS, Synergie 200H). The peak load (N) and energy to rupture (J) of the muscle sample were determined.

### 2.5. Statistical Analyses

**Figure 1:**
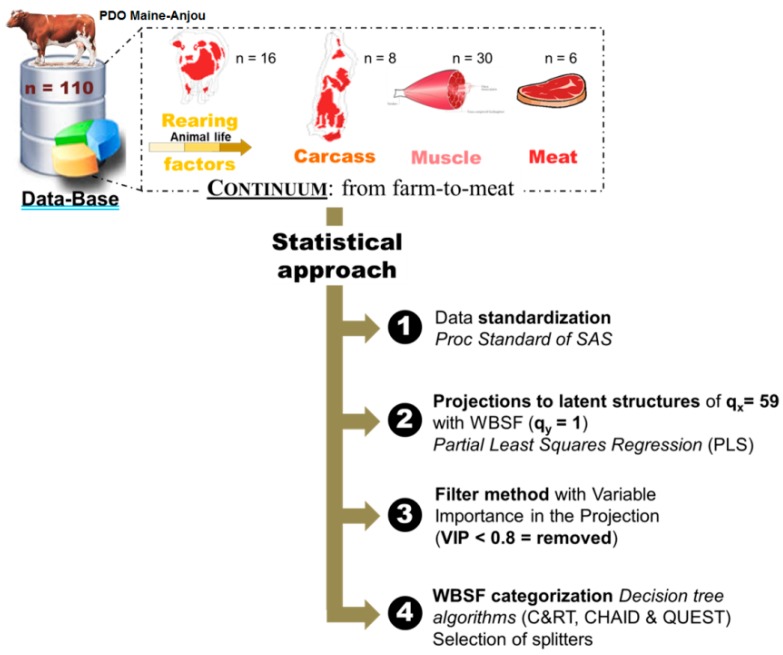
Summary of the statistical approach highlighting the four main statistical steps followed in this study for the selection of best variables from the 59-continuum data from farm-to-meat and then Warner-Bratzler Shear force (WBSF) prediction/categorization into different classes using 3 decision tree algorithms to select the best method.

The data analyses were carried out following 4 main steps as described in Figure 1 using the following statistical software: XLSTAT 2018.2 (AddinSoft, Paris, France) and SAS 9.3 (SAS Institute INC, Cary, NC, USA). A total of 60 variables (q) at 4 levels of the continuum: (i) farm (q_X_ = 16), (ii) slaughterhouse (q_X_ = 8), (iii) muscle (q_X_ = 30) and iv) meat (q_X_ = 5/ q_Y_ = 1) were integrated in this trial (Table 1, Table 2, Table 3 and Table 4). Smirnov-Grubb’s outlier test at a significance level of 5% was first applied for the whole data to check any entry errors or outliers. Subsequently, Shapiro-Wilk tests were applied to determine the normality of data distribution. Descriptive analyses for all the variables were computed (Table 1, Table 2, Table 3 and Table 4). For modeling and before Partial Least Squares (PLS) analyses on q_X_ = 59 variables to explain WBSF (q_Y_ = 1), the data were standardized by computing Z-scores. Z-scores are deviation of each observation relative to the mean of each individual (cow) and amongst each rearing practice [11,22]. PROC STANDARD of SAS was used to standardize the whole data to a mean of 0 and standard deviation of 1. This step allowed removing the effects of rearing practices and variability in the units as well as in the scales among the different variables. It is worthwhile to note that our previous work using the same data showed that rearing practices had no effect on tenderness assessed by trained panelist or instrumental measure by WBSF [11,12].

PLS was then used to identify how the set of explanatory variables (q_X_ = 59) was associated to WBSF (instrumental beef tenderness, q_Y_ = 1) and to select the main driving factors (variables) from each level of the continuum. Briefly, this method consists of relating two data matrices X and Y to each other. In our case, X consists of continuum data except WBSF (X-matrix, 59 variables) and Y is instrumental tenderness measured by WBSF (Y-matrix, 1 variable). The filter method with the variable importance in the projection (VIP) was subsequently used to select the most important variables in the model [23,24]. Thus, the variables with a VIP < 0.8 were all eliminated and the retained variables were used to build decision trees based on the frequently used decision tree algorithms [25]: C&RT (classification and regression tree); QUEST (quick, unbiased, efficient regression tree) and CHAID (Chi-squared Automatic Interaction Detection). This step intends to validate the main variables allowing splitting into three tenderness categories (Tender, Medium and Tough) the beef cuts according to their WBSF values using the whole retained variables in the PLS model. The same criteria of accuracy, sensitivity and specificity used by Gagaoua et al. [25] were applied in this data to choose the best decision tree method. Therefore, the best decision tree was obtained by CHAID method. The identified tenderness groups were further separated by variance analysis using the PROC GLM of SAS on each splitter retained in the decision tree. Variables were considered significantly different among the tenderness classes at the significance level of *p* < 0.05 using Tukey’s test.

## 3. Results and Discussion

**Figure 2:**
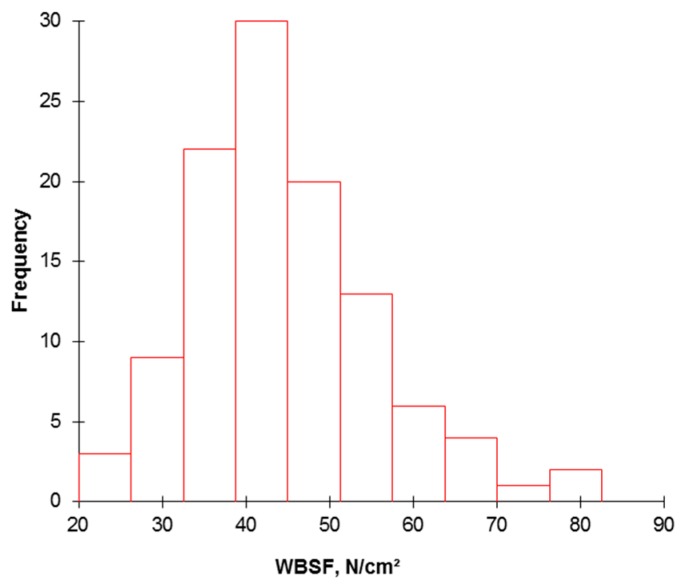
Histogram highlighting the relative frequency for meat tenderness assessed by WBSF on the 110 PDO Maine-Anjou cows.

The descriptive analyses of the data (mean, SD, and minimum and maximum ranges) at each level of the continuum are given in Table 1, Table 2, Table 3 and Table 4. Warner-Bratzler shear force (WBSF) is a routine instrumental measure used as a proxy for sensory testing for meat tenderness. The WBSF values ranged from 23.55–81.49 N/cm^2^, with an average of 44.6 N/cm^2^ (SD 11.21 N/cm^2^). The coefficient of variation was, therefore, 25.1%. This indicates a high variability in tenderness of the population of PDO Maine-Anjou (Figure 2). This was reported by previous studies [26,27,28,29,30] and is a common result in the field of meat texture quality. However, there is scarcity in the studies available on meat tenderness of PDO Maine-Anjou breed rather than this database to perform any comparisons.

The best WBSF PLS model retained 24 variables to explain tenderness variability (Table 5). From the whole 59 explanatory (independent) variables included in the PLS model, 35 had variable importance in the projection (VIP < 0.80) and were removed based on the filter method (Figure 1). This step improved the variation explained in the second model (R^2^X: from 0.17–0.31) and the powerful of the link (R^2^Y: from 0.37–0.64) with the dependent variable that is WBSF. The final model explained 75% of the variability of WBSF (Table 5). Among the 24 variables, six were from farm level (age of weaning; grass diet, %; haylage diet, %; birth month; type of animal (meat or dairy) and physical activity at farm, %), four from slaughterhouse level (color and tenderness scores of the carcasses; ribeye weight and EUROP conformation score), 11 were from the muscle level and mostly they were tenderness protein biomarkers (fiber area, µ-calpain, m-calpain, SOD1, ICDH, DJ-1, PGM1, HSP70-8, LDH, and total and insoluble collagen) and three were from meat level (pHu, redness (a*) and yellowness (b*)). The ranks of each of the 24 variables in the model based on their VIP are further given in Table 5.

**Figure 3:**
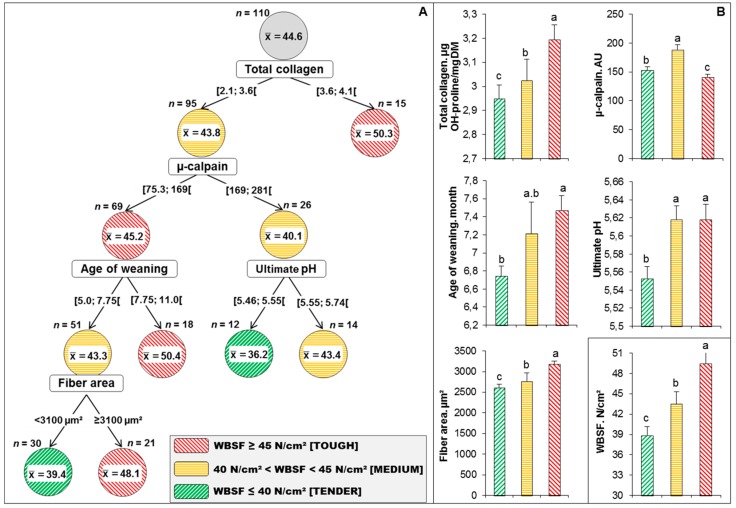
Categorization of the 110 steaks into different tenderness categories based on the decision tree. (**A**) Best decision tree obtained by the Chi-squared Automatic Interaction Detection (CHAID) method built using the list of variables retained in Table 5 to predict correctly 69.4% of WBSF values into three tenderness categories (Red: Tough meat; Orange: Medium meat; Green: Tender meat). The distribution of the animals in each WBSF cluster was used for accuracy measurement. At the beginning of the decision tree, all of the data (*n* = 110) are concentrated at a root node located at the top of the tree. This was then divided into two child nodes on the basis of an independent variable (1st splitter = total collagen), that creates the best homogeneity. The cut-off value of each dividing splitter was calculated from the data of all the subjects. Therefore, the data in each child node are more homogenous than those in the upper parent node. This process is continued repeatedly for each child node until all of the data in each node have the greatest possible homogeneity. This node is called a terminal node and no more branches are possible. (**B**) Variance analysis on the variables retained by the CHAID decision tree among the three WBSF (tenderness) categories that were all significant at *p* < 0.05 (Tukey’s test). The mean values of WBSF among the three tenderness categories were further given at the bottom right of the graph B. Least-square means in the same graph with different superscript letters (a–c) are significantly different (*p* < 0.05).

The objective with respect to implementing decision tools is to propose a model that accurately explains beef tenderness by learning simple decision rules inferred from the individual values of WBSF using the retained potential predictors from Table 5. Accordingly, the best decision tree built using the retained variables in the PLS model was that of CHAID method (Figure 3). CHAID is a recursive partitioning based on the χ^2^-test, which is used to select the best split at each step [31]. Briefly, a CHAID tree is a decision tree that is built by repeatedly splitting subsets of the space into two or more child nodes, beginning with the first whole dataset [32]. To define the best split at any node of decision tree, any allowable pair of categories of the independent variables is merged until there is no statistically significant difference within the pair with respect to the target variable. CHAID has also the peculiarity to proceed stepwise, thus being more adept at handling interactions between explanatory variables, which are available from an examination of the tree. Then, the final nodes of the tree identify subcategories determined by different sets of explanatory variables. The WBSF CHAID decision tree in this trial (Figure 3A) has 4 levels and 11 nodes out of which 6 are considered as terminal (they do not split further). From the 6 terminal nodes, the tree allowed identifying 3 different classes of WBSF (differing in their tenderness: Tender (2 nodes), Medium (1 node) and Tough (3 nodes)) using 5 splitting variables only. The resultant model yielded an overall predictive accuracy of 69.4% compared to the raw data of each individual observation of WBSF.

From the five splitters, three were the first drivers of the PLS model (highlighted in bold character in Table 5). The first splitter was total collagen and generated two groups. As expected, the 15 steaks of the first node (right of decision tree) with total collagen ≥ 3.6 μg OH-proline/mg had the highest WBSF values (mean value = 50.3 N/cm^2^) and considered as Tough meat [27,33]. After that, the second group (*n* = 95) was clustered by µ-calpain at a threshold of 169 AU. The group on the right (*n* = 26) was then separated by ultimate pH at a threshold of 5.55 into 14 medium steaks (WBSF mean value = 43.4 N/cm^2^) and 12 very tender steaks (WBSF mean value = 36.2 N/cm^2^). The group on the left (*n* = 69) was separated by the age of weaning of the animals into a final tough group (WBSF ≥ 45, *n* = 18) and a medium group of 51 steaks, which were then categorized by fiber area at a threshold of 3100 µm^2^ into 30 tender (WBSF mean value = 39.4 N/cm^2^) and 21 tough steaks (WBSF mean value = 48.1 N/cm^2^). The mean values of WBSF among the three tenderness categories are 38.9 ± 8.1 N/cm^2^, 43.4 ± 6.6 N/cm^2^ and 49.4 ± 12.0 N/cm^2^ for tender, medium and tough steaks, respectively. Therefore, the CHAID decision tree could simply and easily apply the discrimination rule based on five splitters to identify these three tenderness categories.

It seems from the whole data of the continuum based on the PLS model and CHAID decision tree that muscle characteristics (from the muscle level) namely total collagen content, µ-calpain and fiber area, are the main potential discriminators/predictors of tenderness of PDO Maine-Anjou (Figure 3B). Collagen is well known to be associated with the background of meat toughness [34,35]. In a meta-analysis on the main parameters affecting collagen amount and its heat-solubility, Blanco et al. [36] hypothesized that total collagen content is different among muscles but with high amounts at birth, thereafter decreasing from birth to puberty as a function of muscle growth [35]. They further linked their model to proteolysis, in line to the involvement of µ-calpain, cross-sectional area of the fibers and age of weaning of the animal. In a recent metadata of 308 young bulls that were categorized into three tenderness classes, total collagen was a discriminator of the built tenderness classes with a mean value for the tough class of 3.51 µg OH-prol·mg^−1^ DM. These findings agree with the negative link of collagen with tenderness evaluated by trained panelist or instrumental, as reported in numerous studies [13,14,17,37] and reviewed by Lepetit [38,39].

The two other variables are related to farm level (rearing practices) by age of weaning or at the meat level by ultimate pH. Accordingly, the decision tree (Figure 3A) allowed to identify that a steak of the PDO Maine-Anjou was considered tender (lowest WBSF: < 40N/cm^2^) if it matched the following two rules:

(i) IF (total collagen < 3.6 μg OH-proline/mg) AND (µ-calpain ≥ 169 AU) AND (ultimate pH < 5.55) THEN meat was very tender (mean WBSF values = 36.2 N/cm^2^, *n* = 12); or
(ii) IF (total collagen < 3.6 μg OH-proline/mg) AND (µ-calpain < 169 AU) AND (age of weaning < 7.75 months) AND (fiber area < 3100 µm^2^) THEN meat was tender (mean WBSF values = 39.4 N/cm^2^, *n* = 30).

In addition to the predictive rules allowed by the statistical approach applied in this study, possible biological mechanisms behind meat tenderness determinism of PDO Maine-Anjou breed are further revealed. It seems that the final tenderness of this breed is mainly related to the extent breakdown of structural properties in the muscle and to the background toughness related to connective tissue. This is in agreement with the recent findings of a proteomic study based on a sub dataset of eight PDO Maine-Anjou cows categorized into four tender (WBSF < 31 N/cm^2^) and 4 tough (N > 60 N/cm^2^) samples [40]. In this study, the authors identified eight potential biomarkers explaining differences in tenderness, of which six are structural proteins. The interesting links allowed by the decision tree within pH drop presented by ultimate pH and proteolysis by the abundance of µ-calpain support these findings. Furthermore, these are consistent with several studies from the large literature [41,42,43,44]. For example, an earlier work by Dransfield et al. [45] reported strong relationship between glycolysis and μ-calpain activity with a strong effect on final tenderness of meat.

The involvement of age at weaning and the cross-sectional area of the fibers in the prediction of tenderness may be explained by two reasons. First, it is known that animal growth affect muscle development and consequently its composition including connective tissue [46], evaluated in this study by collagen content and solubility. Second, animal growth had important consequences on protein turnover know to influence the final qualities of meat [47]. Indeed, previous studies have reported significant relationships between the speed of development of the animal and therefore its carcass composition with tenderness of cows according to age at weaning [48]. Overall, these results allow us to propose the PLS—CHAID decision trees as an interesting tool for validation on other types of animals and other qualities of meat for use by the farmers as well as the slaughterers in order to classify (or predict) the potential quality of carcasses soon after slaughter.

## 4. Conclusions

The purpose of this trial was to investigate the usefulness of combining chemometrics and machine learning tools to predict tenderness of PDO Maine-Anjou cows. First, we analyzed the potential of Partial Least Squares to select the main variables (or variables of interest) from the continuum to explain WBSF of ribeye steaks. The filter method allowed retaining 24 variables from 59 to explain WBSF variability. Second, using the CHAID decision tree as the best algorithm method among others, the 110 steaks were categorized into three tenderness classes using five splitters: total collagen, µ-calpain, fiber area, age of weaning and ultimate pH. Three of these potential predictors belong mainly to the muscle level and the two last other predictors to the farm and meat levels. The original statistical approach applied in this trial allowed us to properly group steaks for their tenderness potential using variables of the farm-to-meat continuum data. In the future, this proposed approach would be combined with sensory scores of tenderness (using trained panelists or consumers) to improve the prediction power and accuracy as well the validation of the retained splitters as predictors of PDO Maine-Anjou tenderness. Finally, the proposed tool would be further adopted for validation on other animal types and will be proposed for use by the beef sector to accurately categorize carcasses according to their tenderness potential. This would be beneficial at both the economic and consumer levels.

## Figures and Tables

**Table 1:** Average values and variations of the data from the farm level describing the 16 variables of animal characteristics and finishing period ^1^.

Variables	n	Mean	SD	Min	Max
Birth weight (kg)	100	49.9	4.91	38	66
Month of birth (1–12)	110	-	-	1	12
Genetic type (0: Beef or 1: Dairy)	110	-	-	0	1
Age of weaning (month)	107	7.2	1.07	5	11
Weaning duration ^2^	110	8.7	9.41	0	36
Age at first calving (month)	110	32.4	4.09	18	43
Number of calving	110	3	2.05	1	9
Suckling score (0–10)	103	5.9	1.36	3	9
Fattening duration (day)	110	98.6	29.96	37	203
Haylage diet (%)	110	27.8	36.98	0	100
Hay diet (%)	110	48.2	37.39	0	100
Grass diet (%)	110	24	32.1	0	100
Daily concentrate diet (kg)	110	7.7	2.13	2	13
Global concentrate diet (kg)	110	738	244	178	1330
Activity (%)	110	54	46.21	0	100
Age at slaughter (month)	110	67.5	24.79	34	120

^1^ These data were obtained for each individual cow following the survey described in the questionnaire conducted by Couvreur et al. [12] including information about the finishing period and animal characteristics. ^2^ Represent the period between the last weaning and the beginning of the fattening period (days).

**Table 2:** Average values and variations of the data from the slaughterhouse level describing the eight carcass characteristics.

Variables	n	Mean	SD	Min	Max
Carcass weight (kg)	110	438.2	36.09	380	553
Conformation score (1–15 scale) ^1^	107	7.8	0.82	6	10
5th rib weight (g)	110	3079	638	1793	5640
Muscle carcass weight (g) ^2^	110	1882	403	1145	3478
Fat carcass weight (g) ^2^	110	582	190	216	1338
Fat-to-muscle ratio in the 5th rib (% *w*/*w*)	110	31.3	10.17	16	85
Color score of the carcass (1–5) ^3^	105	2.9	0.38	2	4
Tenderness score of the carcass (1–5) ^4^	105	3.4	0.65	2	5

^1^ EUROP classification grid for carcass conformation scores from P− = 1 to E+ = 15. ^2^ Muscle and fat carcass weights were estimated after dissection of the 5th rib as Gagaoua et al. [7]. The equations used are described in detail in the study by Couvreur et al. [12]. ^3^ Visual score assessing meat color from 1–5 was evaluated by the same experts familiar with the EUROP grid according to the PDO Maine-Anjou agreement. ^4^ Palpation of the 5th rib allowed determining on 1–5 scale the tenderness potential of the steaks.

**Table 3:** Average values and variations of the data from the muscle level describing the 30 quantified characteristics in *Longissimus thoracis* muscle including protein biomarkers for the 110 cows.

Variables	Mean	SD	Min	Max
**a. Contractile properties by myosin fibers characterization**				
Fiber area. µm^2^	2906	646	1762	5203
MyHC-I, %	31.2	7.37	15.22	69
MyHC-IIa, %	56.6	12.78	23.76	84.78
MyHC-IIx/b, %	12.2	14.03	0	53.91
**b. Metabolic properties by metabolic enzyme activities**				
LDH (μmol·min^−1^·g^−1^)	1.05	0.33	0.31	2.26
ICDH (μmol·min^−1^·g^−1^)	703	109	491	939
**c. Intramuscular connective tissue properties**				
Total collagen μg OH-prol·mg^−1^ DM	3.1	0.42	2.08	4.06
Insoluble collagen μg OH-prol·mg^−1^ DM	2.4	0.33	1.61	3.26
Soluble collagen %	20.8	2.94	14.85	26.58
**d. Protein biomarkers quantified by Dot-Blot (in arbitrary units)**				
Heat shock proteins				
CRYAB	226.4	83.96	59.04	576.89
Hsp20	164.8	45.45	59.84	306.74
Hsp27	79.7	19.83	36.88	134.56
Hsp40	130.5	20.97	96.09	280.56
Hsp70-1A	111.4	24.81	61.29	180.36
Hsp70-1B	120.1	26.16	70.38	187.36
Hsp70-8	184.5	49.43	50.12	432.19
Hsp70-Grp75	144.5	30.5	87.12	213.24
Metabolism				
Enolase 3 (ENO3)	144.3	36.22	78.74	258.12
Phosphoglucomutase 1 (PGM1)	101	27.26	46.88	254.36
Structure				
α-Actin	122.7	40.37	56.99	266.14
Myosin binding protein H (MyBP-H)	90.2	27.49	42.05	184.32
Myosin light chain 1F (MyLC-1F)	63.8	12.91	33.23	91.06
Mysoin heavy chain IIx (MyHC-IIx)	124.9	18.55	80.91	182.28
Oxidative stress				
Superoxide dismutase [Cu-Zn] (SOD1)	101.5	37.92	23.95	167.44
Peroxiredoxin 6 (PRDX6)	106.2	17.41	73.78	163.74
Protein deglycase (DJ1)	90.6	13.9	58.12	146.92
Proteolysis				
µ-calpain	151.7	38.24	75.28	281.08
m-calpain	96.1	12.62	64.69	124.75
Apoptosis and signaling				
Tumor protein p53 (TP53)	118.3	22.31	78.36	175.78
H2A Histone Family Member X (H2AFX)	98.7	19.01	58.72	153.83

**Table 4:** Descriptive statistics of the 6 variables from the meat level corresponding to meat quality traits measured in *Longissimus thoracis* muscle.

Variables	n	Mean	SD	Min	Max
Warner-Bratzler shear force (N/cm^2^)	110	44.6	11.21	23.55	81.49
Intramuscular fat (IMF) content (% *w*/*w*)	110	16.3	6.18	6.15	40.34
Ultimate pH (pHu)	107	5.6	0.1	5.34	6.22
Lightness (L*)	110	39.7	2.3	34.36	46.84
Redness (a*)	110	8.8	1.24	4.17	11.77
Yellowness (b*)	110	7.4	1.43	4.02	11.42

**Table 5:** Best Partial Least Squares (PLS) model of Warner-Bratzler Shear force (WBSF) showing the ranking of the 24 retained variables from the continuum data from farm-to-meat and their variable importance in the projection (VIP) values.

Variables of the Continuum from Farm-To-Meat Data	Rank	VIP
**Farm level:** rearing factors and animal characteristics		
Age of weaning, month	3	1.99
Grass diet, %	10	1.31
Haylage diet, %	14	1.12
Birth month	15	1.11
Type of animal (meat or dairy)	16	0.97
Physical activity at farm, %	24	0.84
**Slaughterhouse level:** carcass characteristics		
Color score, 1–5 scale	5	1.8
Carcass tenderness score, 1–5 scale	21	0.9
Ribeye weight, g	20	0.94
EUROP Conformation score, 1–15 scale	23	0.87
**Muscle level:** protein biomarkers		
Fiber area, µm^2^	2	2.01
SOD1, AU	4	1.94
m-calpain, AU	6	1.64
ICDH, μmol·min^−1^·g^−1^	7	1.57
Protein deglycase (DJ-1), AU	9	1.51
PGM1, AU	11	1.27
Insoluble collagen, μg OH-proline/mg DM	13	1.18
HSP70-8, AU	17	0.97
µ-calpain, AU	18	0.96
Total collagen, μg OH-proline/mg DM	19	0.96
LDH, μmol·min^−1^·g^−1^	22	0.89
Meat level: meat quality traits		
pHu	1	3.29
Redness (a*)	8	1.53
Yellowness (b*)	12	1.27
